# Editorial: Milk oligosaccharides and lipids, their impact on human health

**DOI:** 10.3389/fnut.2023.1255077

**Published:** 2023-09-15

**Authors:** Yajun Xu

**Affiliations:** ^1^Department of Nutrition and Food Hygiene, School of Public Health, Peking University, Beijing, China; ^2^Beijing Key Laboratory of Toxicological Research and Risk Assessment for Food Safety, Peking University, Beijing, China; ^3^Peking University Health Science Center, China Feihe Joint Research Institute of Nutrition and Healthy Lifespan Development, Beijing, China

**Keywords:** milk oligosaccharides, lipids, human health, milk, nutrition

The Research Topic collects researches on the contents, composition and physiological effects of milk oligosaccharides and lipids milk with the aim of deepening the understanding of the composition of milk and its impacts on human health, and promoting its application in the field of life health. A total of four original researches on the above-mentioned topics have been published in the Research Topic.

Human milk (HM) is a huge treasure trove containing macronutrients and micronutrients, and a large number of active ingredients and supplies sufficient energy, nutrition and effective protection for infants. HM has always been considered as the best food for infants in the first 6 months of life. Human milk oligosaccharides (HMOs) are the third most solid components in milk secondary to lactose and fat, and have attracted extensive attention due to their multiple physiological functions such as effective prebiotics, anti-inflammatory effects and regulating immune. The HMO profiles in HM vary greatly from individual to individual, and are influenced by a series of factors including genetic background, maternal related factors, geographical environment and diet. Genetic background partly shaping the composition and contents of HMOs has been confirmed by most studies. However, there are not sufficient systematic studies on the impacts of other environmental factors on HMOs spectrum. In the Research Topic, Fan et al. systematically analyzed how maternal factors such as age, race, feeding methods, and dietary intake affected HMOs profiles. Researchers found that except for 3′-Sialyllactose (3′SL) and 6′SL, 72% of participants were “secretor mothers” who had distinct HMOs contents and composition from those “non-secretor mothers.” Maternal body weight, pregnancy complications and diet had significant impacts on HMOs. The contents of 3′SL in HM of mothers with pre-pregnancy overweight/obese increased significantly. In non-secretor mothers, LNPF I + LNPF III abundances were greater in overweight mothers than those in normal weight mothers. As for dietary factors, the consumption of egg had a significantly positive association with LNT+LnNT contents, and 2′-Fucosyllactose (2′FL) and S-LNnH II showed the increasing trend with the increment of maternal cheese intake. The findings indicate that improving maternal nutrition and health status is critical for the refinement of HMOs profiles in HM, and provide the potential intervention target for maternal and infant care and health.

The huge structural variability and tremendous kinds impede the large-scale commercial production and application of HMOs to some extent. However, 2 'FL, one of the most abundant HMOs in HM, has been widely used in maternal and infant field. The infant formula supplemented with 2 'FL has been officially approved in several countries including the European Union and the United States. The infant formula with 2′FL presents the beneficial effects on the gut health and mature, the intestinal microecology, and the prevention of infectious diseases in artificially-fed infants, and the growth and development outcomes even reach a level similar to that of breast-fed infants (1). In addition to promoting local intestinal health, 2 'FL also plays a positive role in the distant organ health. In the Research Topic, Gart et al. found that 2′FL could inhibit the expression of genes involved in endoplasmic reticulum (ER) stress (ATF4, ATF6, ERN1, and NUPR1) and reduce diacylglycerols in liver, thereby decreasing the level of circulating insulin and correct liver insulin resistance. Furthermore, 2′FL also promoted the expression of ACOX1 gene related to lipid synthesis, and caused the down-expression of SREBF1 involved in lipid synthesis, which avoided the occurrence of non-alcoholic fatty liver disease (NAFLD). The findings expand our understanding of the physical functions of 2′FL, and provide evidence support for the clinical application of 2′ FL.

Protein is the fourth component of HM. The glycosylation of proteins is an important post-translational modification, which exerts active effects on protein functions in HM. Compared to proteins in HM, the glycoproteins were abundant in cell adhesion, proteolysis, and defense/immune process. Lu et al. identified 998 proteins and 764 glycosylated sites from 402 glycoproteins in HM by using TNT labeling proteomics. The glycosylated sites changed along the lactation. In colostrum, 78 glycosylated sites from 56 glycoproteins significantly increased, and 10 glycosylated sites from 10 glycoproteins were higher in the mature HM. Additionally, these differentiated glycoproteins were mainly involved in host defense. Furthermore, it was intriguing to find that one glycosylated site (Asp144) in IgA and two glycosylated sites (Asp38 and Asp1079) in tenascin were significantly upregulated while their protein abundance was reduced during lactation. Researchers figured out the glycosylated sites in glycoproteins and revealed their dynamic changes during the lactation as well as related functions. The findings promote the understanding of composition of HM, which is conducive to the development of food for infant's health.

Lipids are main energy source in HM and provide 40–50% of daily energy demand of infants. Triglyceride (TG) is the major form of fat in HM, and the difference in HM lipids lies in the varied fatty acids (FAs) making up triglycerides. Palmitic acid (PA) (C16:0) shows the highest content of saturated fatty acids (SFAs) in HM, accounting for about 3% of all FAs. The binding site of PA in TG is located at *sn-2* (2). This specific location of PA contributes to the absorption of FAs and the improvement of mineral balance in gut, which is also an important reason why HM is significantly superior to milk of other mammals (Chen et al.). As for unsaturated fatty acids, monounsaturated fatty acids (MUFAs) and polyunsaturated fatty acids (PUFAs) accounted for 36.6 and 21.0% of total fat in HM, respectively. Oleic acid [18:1n-9 (n-9)] is predominant among MUFAs and n-3 PUFAs have a relatively higher contents, among which alpha-linolenic acid (ALA), eicosapentaenoic acid (EPA), and docosahexaenoic acid (DHA) are essential and abundant. N-3 PUFAs are vital to the nervous system, visual, motor and cognition development of infants. Lipids produced within the secretory cells of the mammary gland are encapsulated by the milk fat globule membrane (MFGM) that is a three-layer phospholipid molecular layer structure (3). The progress of producing MFGM means that glycoproteins, carbohydrates and lipids from mothers enter into HM, which acts as the mediator linking mothers to infants. Currently, the physiological functions of MFGM are mainly manifested in its positive impacts on infants' cognition, immune regulation, intestinal development, and metabolism.

HM is the optimal food choice for infants, and milk is the vital component of diets among general population. It has been reported that the annual per capita consumption of dairy products increased by 36.3% in the past decade in China (4). The components in milk positively or negatively affect human health, which is partly related to the composition of FAs in milk. There are over 400 kinds of FAs in milk, among which UFAs tend to be beneficial for the cardiovascular diseases (CVD), while SFA is commonly regarded as a detrimental factor for CVD. However, the current study showed that odd- and branched-chain fatty acids (OBCFA) in milk present positive effects on CVD, which might be explained by the structural differences and complexity. Therefore, it is important to identify comprehensively FAs' structure and profiles for the evaluation of health effects of FAs in milk. In the Research Topic, Chen et al. developed gas chromatography-mass spectrometry (GC-MS) to test retail bovine milk in China and determine 11 even-chain saturated FAs, 10 odd-chain saturated FAs, nine branched-chain saturated FAs, 30 monounsaturated FAs, and 22 polyunsaturated FAs, a total of 82 FAs. The findings updated the FA profiles in retail bovine milk in China and provide the reference for the refinement FA composition in milk and the recommendation of daily intake of milk.

In this Research Topic, focusing on oligosaccharides and lipids, as two typical components in milk, the composition profiles of milk and its effects on health are figured out, and these findings are of great scientific significance. The findings on HMOs and glycoproteins in HM promote the further understanding of the composition of HM and the revelation of its health effects, and more favorable scientific evidence is provided for promoting breastfeeding and achieve the healthy development of mothers and children. Additionally, it helps optimizing the composition of infant formula, which ensures that artificially-fed infants who cannot be breastfed obtain high-quality nutrition like breastfed infants. These findings also remind us that although research on the composition of HM has made amazing progress, we still cannot completely uncover the mystery of the HM, which constantly drives the conduction and deepening of the exploration of HM. Moreover, FA profiles in milk provides us with a new insight of health effects of bovine milk and better guide the daily intake of milk.

## Author contributions

YX: Writing—review and editing.
